# Survival strategies of artificial active agents

**DOI:** 10.1038/s41598-023-32267-3

**Published:** 2023-04-06

**Authors:** Luigi Zanovello, Richard J. G. Löffler, Michele Caraglio, Thomas Franosch, Martin M. Hanczyc, Pietro Faccioli

**Affiliations:** 1 Physics Department, University of Trento, Via Sommarive 14, Povo, Trento 38123 Italy; 2 Institut für Theoretische Physik, Universität Innsbruck, Technikerstraße 21A, 6020 Innsbruck, Austria; 3 Department of Cellular, Computational and Integrative Biology (CIBIO), University of Trento, Via Sommarive 9, Povo, Trento 38123 Italy; 4 Department of Chemical and Biological Engineering, University of New Mexico, Albuquerque, NM 87106 USA; 5 Trento Institute for Fundamental Physics and Applications (INFN-TIFPA), Via Sommarive 14, Povo, Trento 38123 Italy

**Keywords:** Chemical physics, Statistical physics, Biophysical chemistry, Supramolecular chemistry

## Abstract

Artificial cells can be engineered to display dynamics sharing remarkable features in common with the survival behavior of living organisms. In particular, such active systems can respond to stimuli provided by the environment and undertake specific displacements to remain out of equilibrium, e.g. by moving towards regions with higher fuel concentration. In spite of the intense experimental activity aiming at investigating this fascinating behavior, a rigorous definition and characterization of such “survival strategies” from a statistical physics perspective is still missing. In this work, we take a first step in this direction by adapting and applying to active systems the theoretical framework of Transition Path Theory, which was originally introduced to investigate rare thermally activated transitions in passive systems. We perform experiments on camphor disks navigating Petri dishes and perform simulations in the paradigmatic active Brownian particle model to show how the notions of transition probability density and committor function provide the pivotal concepts to identify survival strategies, improve modeling, and obtain and validate experimentally testable predictions. The definition of survival in these artificial systems paves the way to move beyond simple observation and to formally characterize, design and predict complex life-like behaviors.

## Introduction

Natural biological systems are capable of developing strategies to survive and reproduce, forming the basis for the process of Darwinian evolution^1^. Artificial life aims at recreating traits of these systems to gain a deeper understanding of the complex information processing that defines biological phenomena and evolution^2,3^. As a part of this approach, several recent efforts to produce artificial cells with widely varying components, but often with pieces isolated from natural living cells, have shown fascinating dynamics. In particular, they can respond dynamically to stimuli when away from thermal equilibrium, e.g. through self-division^4^. Some synthetic systems can also execute specific patterns of moves that ultimately result in the delay of the eventual approach to chemical equilibrium^5^. A notable example is the motile chemical droplet^6–8^. In this specific case, a liquid droplet is charged with chemical potential in the form of fuel that is consumed during the reaction. Motion is the emergent mechanism or behavior that slows the droplets eventual decay towards equilibrium^6^.

From a statistical physics perspective, being out-of-equilibrium represents the most general feature that these artificial agents have in common with biological organisms^9^. For this reason, in the following, we shall say that a synthetic agent is also “surviving” if it is not in equilibrium with its environment. The survival of an agent is typically associated with specific features of its behavior, such as e.g. the migration towards regions with higher concentration of nutrients (fuel) or away from regions with a high concentration of products resulting from fuel consumption (waste). In this sense, the quest for survival is closely related to a target-search problem^10–17^.

The observation that active agents are capable of undertaking specific target-search motion ultimately determining their survival raises a number of questions: Do the survival “strategies” undertaken by a given active agent exhibit common universal features or are they intrinsically heterogeneous and specific to the characteristics of the agent itself? To what extent are the survival strategies determined by the initial conditions? Is it possible to assign a probability for an artificial agent or a biological cell initially placed in a specific location to ultimately survive on a long time scale? In this work, we set the stage to address these fundamental questions from a physics-based, statistical mechanical perspective.

To define, characterize, and predict the survival strategies undertaken by an active agent, we expand to nonequilibrium conditions some concepts and notions of Transition Path Theory, the classical statistical–mechanical theory describing rare spontaneous stochastic transitions between metastable states^18–22^. In particular, we resort on the pivotal concept known as the committor function^19,22–26^, which has already been successfully exploited to investigate long-range correlations and nucleation pathways in active-matter systems^26,27^. In general terms, this quantity, encoding the probability for a stochastic system to reach a predetermined goal as a function of the initial conditions, has been widely exploited in the framework of chemical physics^22,28^, as it was shown to provide an ideal reaction coordinate for thermally activated transitions of passive systems, such as e.g. structural rearrangements of biomolecules^29,30^. In this context, it is defined as the probability for the molecule prepared in some initial configuration to reach a given product state (e.g. for a protein, its native structure) without first backtracking to a competing state (e.g. the protein unfolded state).

To address our questions concerning survival strategies in artificial systems, we consider the classical example of a camphor disk in water or, to be more precise, an improved camphor–camphene–polymer disk capable of long-term self-propulsion^31,32^. We also consider one of the simplest paradigmatic theoretical models of active stochastic dynamics, namely the active Brownian particle (ABP)^33,34^. This model is obtained through a generalization of the Brownian dynamics with the addition of a self-propulsion speed with variable orientation that keeps the system out of equilibrium (see “Materials and methods” section). Both the camphor disk and the ABP navigate in a circular enviroment in which particular sub-regions affect differently their surviving odds. By comparing the target-search strategies obtained by simulations of ABPs to experimental trajectories of camphor disks, similar qualitative behaviors of the committor emerge, thus unveiling features of survival strategies which depend on the particular environment but are independent of the specific agent. Furthermore, looking at the ABP as a first tentative model for the camphor disk dynamics, an analysis of the committor and of other typical quantities of Transition Path Theory provides direct assessment of the model validity and suggestions on its improvement.

## Results and discussion

### Statistical physics model of survival dynamics

The camphor disk moves in a two-dimensional environment given by the Petri dish and, at each time *t*, the system’s configuration is specified by its instantaneous position $\varvec{r}(t)=(x(t),y(t))$ and velocity $\varvec{v}(t)=(v_x(t),v_y(t))$^31,32^ (see also “Materials and methods” section). The same holds for an ABP with the difference that, in this case, the modulus of the self-propulsion velocity, *v*, is fixed and only its direction $\vartheta (t)$ explicitly appears in the definition of the system’s configuration (see “Materials and methods” section). On the other hand, the state of a passive Brownian particle, used as a reference system, only includes the instantaneous position $\varvec{r}(t)$. More generally, we then denote with $\Gamma (t)$ a system’s microstate and with $X_t = \{\Gamma (0)\rightarrow \Gamma (\Delta t) \rightarrow \Gamma (2 \Delta t) \rightarrow \cdots \rightarrow \Gamma (t = n\Delta t)\}$ the whole “history” of the system up to time *t*, i.e. a time-ordered sequence of microstates $\Gamma$ visited, starting from some initial state $\Gamma (0)$ at the initial time $t=0$.

With real-life situations in mind, we bundle the environment in three different disjoint regions (T, R, and Q) which affect differently the surviving odds of the agent. Adopting for convenience a terminology commonly used in the description of reactive processes, the “target” region T is a portion of configuration space with abundance of fuel or nutrients. In the following, we shall also refer to it as the “safe zone” , because in T a out-of-equilibrium condition can persist for time intervals much larger than the characteristic time scale of the system. In contrast, the reactant region R is a “death zone”, characterized by absence of fuel or nutrients or the abundance of consumption waste or poisoning substances. In R, the system rapidly thermalizes with the bath and becomes passive. Finally, in the transition region Q (anywhere outside both T and R) the agent does not risk rapid thermalization but is also unable to sustain activity for a very long time, for example because of limited resources.

We now define a *survival move* as any local transition that does not lead the agent to visit region R or brings the agent out of it. Then, we define a *surviving path* associated with an initial condition $\Gamma _0$ as a stochastic trajectory $X_t$ initiated in $\Gamma _0$ and composed by a sequence of survival moves.

In essence, the survival path ensemble is a natural adaptation to active systems of the concept of the transition path ensemble, which is commonly used to describe rare thermally activated transitions in passive systems. The information about survival “strategies” is encoded in the survival path ensemble, and can be decoded by analyzing a set of stochastic descriptors (distributions and probability fields), which characterize the system’s dynamics.

In particular, the configurations that are most likely visited by the surviving particles can be inferred by computing the local surviving-path density $m_S(\Gamma |\Gamma _0)$, defined as the probability that a surviving trajectory $X_t$ initiated at $\Gamma _0$ visits the microstate $\Gamma$. The $m_S$ distribution allows for important insight about the dynamics of surviving particles. However, it does not provide detailed information on the sequence of moves ultimately leading to survival. It also does not allow computing the probability of survival, given the initial conditions. To provide these missing pieces of information, we rely on the notion of the committor function, which for any point in Q gives the probability of reaching the safe zone before the death zone (mathematical properties of the committor function for passive and active particles are discussed and compared in the Materials and Methods section). In this sense, the committor function coincides with a survival probability and it enables us to obtain a predictive mathematical characterization of the survival strategy.

### Illustration and comparison with experiment

To illustrate our statistical physics scheme and to assess its predictive power, it is instructive to compare theoretical predictions obtained from numerical simulations with the results of experiments performed on artificial active agents. In particular, we have used self-propelled camphor disks floating on a water surface and exploring a confining environment provided by a Petri dish of radius $\sigma$ (more details are provided in the “Materials and methods” section).

We focus our analysis on three different initial conditions, with positions located in small circular regions, denoted by 1, 2, and 3, respectively.

**Figure 1:**
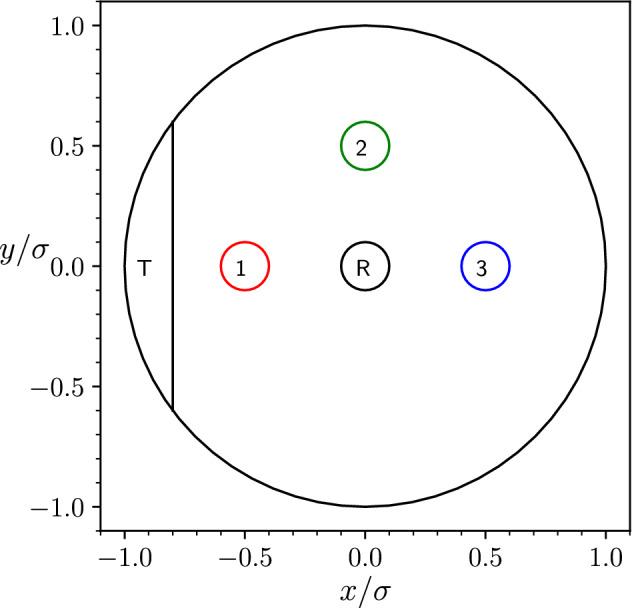
Experimental and simulation setup. The death zone R is a circular region at the center of the Petri dish, while the safe region T is a circular segment on the left side of the dish. The committor is evaluated in the three circular regions labeled from 1 to 3. Here (and in the following) R and T refer respectively to the projections on the physical space of the reactant R and target T which are generally defined in the whole configuration space. The shape, position, and size of regions R and T in the Petri disk where arbitrarily chosen in order to ensure that the system’s configuration does not display any symmetry. The size of the R and T regions have been chosen large enough to guarantee the collection of sufficient statistics in simulations and in our experiments with camphor disks.

#### Survival-path density

As a first step in our analysis, we focus on the survival-path density associated with initial conditions in the three small regions of the Petri dish. For sake of simplicity, we choose to marginalize this distribution with respect to the angular orientation, i.e. to compute

1 $$\begin{aligned} m_s(\varvec{r}|\varvec{r}_a):= \int \textrm{d}\vartheta _0 \int \textrm{d}\vartheta \, m_s(\varvec{r}, \vartheta |\varvec{r}_a, \vartheta _0) \;, \qquad (a=1,2,3). \end{aligned}$$

This distribution can be estimated from a statistical analysis of long stochastic trajectories obtained in simulations (by integrating numerically the ABP stochastic equations of motion). However, differently from the ABP model, in experiments we are not able to directly measure an internal angular variable $\vartheta$ specifying the direction of their self-propulsion velocity. Then, in this case, the survival-path density is simply computed by a frequency histogram of the positions visited by the agent, see Supporting Information—SI for additional details.

**Figure 2:**
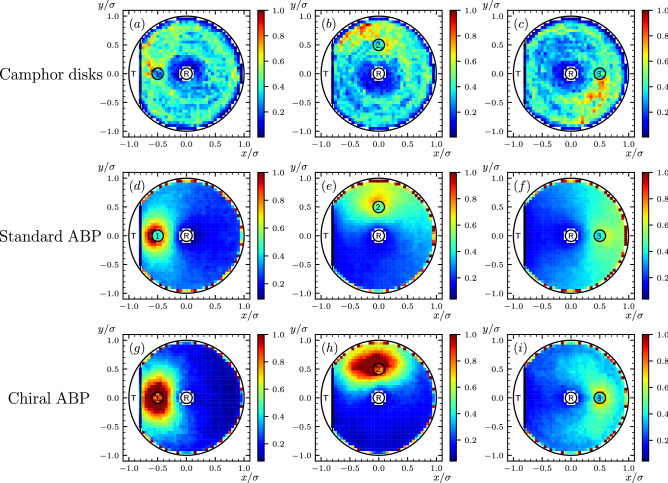
Survival path density $m_s({\varvec{r}}|{\varvec{r}}_a)$—for three different initial conditions ($a=1,2,3$)—extracted from experiment (**a–c**) by tracking the motion of the camphor particles, evaluated from numerical simulation in the standard ABP model (**d–f**), and in the chiral ABP model (**g–i**).

In Fig. 2 we show that the distributions obtained in the plain ABP model share qualitative features in common with those extracted from the experiment.

As expected, these results suggest that the survival paths are likely to visit the portion of the Petri dish located in the direction opposite to the death region relative to the initial condition. This reflects the fact that the chances of survival of a particle initially heading towards the death region are rather low. A less intuitive common feature of theoretical and experimental distributions is the presence of a ring-shaped high survival-path probability density region, near the boundary of the Petri dish. In the theoretical results, this ring is very thin and lies near the edge of the disk. Camphor particles cannot come so close to the boundary, because of finite-size and hydrodynamic effects. Yet, a circle of relatively large survival-path probability density is clearly visible in all panels reporting the experimental results. This feature suggests that a successful survival strategy consists in reaching the target region by sliding along the boundary. This mechanism will be referred to as the “shuttle surviving mechanism” (more below).

The experimentally observed survival strategy displays also a few qualitative differences with respect to the prediction of the standard version of the ABP model. For example, the survival-path distributions obtained from the experimental trajectories display concentric circular “orbits”, a feature that is not captured by the predictions of the standard ABP model. In addition, the experimental results for the second initial condition (panel (b)) are peaked in a region in the upper-left quadrant, while the theoretical ABP distribution is maximum just above the initial position (panel (e)).

**Figure 3:**
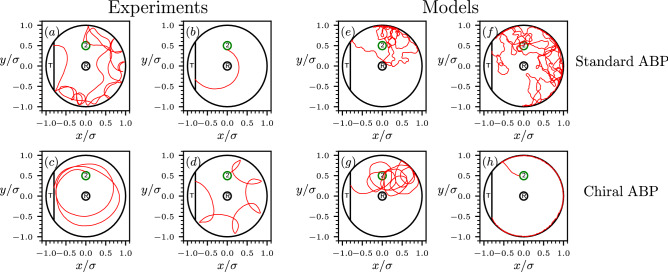
(**a–d**) Typical experimental trajectories of a self-propelled camphor disk reaching the target T starting from the circular region 2. (**e,f**) Typical trajectories of the standard ABP (no angular drift, $\omega =0$). (**g,h**) Examples of trajectories of the chiral ABP.

To further analyze the origin of this observed discrepancies, in Fig. 3 we show a number of different survival trajectories obtained in experiment and we compare them with typical trajectories generated by the simulations in the standard and in the so-called “chiral” variants of the ABP model (defined in “Materials and methods” section). For comparison, in Fig. S3 of SI are reported some typical trajectories reaching the death region before the safe region.

First of all, we note that the experimental pathways display a wide spectrum of variability (see Fig. 3a–d). In particular, while some trajectories are similar to the motion of a standard ABP (Fig. 3a), others present a dominant circular contribution to the motion of the disk (Fig. 3b,c), which in some cases gives rise to “rosette” shaped trajectories (Fig. 3d). This quasi-circular motion is responsible for the emergence of concentric ring-like structures in the survival probability densities (see, e.g., Fig. 2b). The trajectories generated using the standard ABP do not vary considerably, and they show a single type of trajectory shape (see Fig. 3e,f).

The reason for the observed high variability in the experimental trajectories depends on several factors. First of all, the self-propelled camphor disks do not maintain the same level of activity (and thus, the same speed) throughout a single experiment, with a velocity that can vary considerably during the acquisition time. In addition, small asymmetries in their nearly circular shape can emerge spontaneously throughout an experimental run due to an asymmetric loss of the self-propelling fuel or due to hydrodynamic interactions with the boundary. This effect can give rise to asymmetries in the propulsion direction, thus promoting circular trajectories.

The standard ABP model, in contrast, assumes a constant drift velocity and no average rotation of the propulsion direction. Arguably, these features limit the heterogeneity in trajectory shape of standard ABP particles and explain why the chiral ABP model accounts better for the shape variability of the trajectories observed in experiments. In particular, the inclusion of the additional angular drift gives rise to circular trajectories, exemplified in Fig. 3g.

Interestingly, chiral ABP gives rise to circular orbits while preserving a prominent “shuttle” surviving strategy, such as the one reported in Fig. 3h. In fact, depending on whether the angular drift $\omega$ is pushing the particle’s orientation towards the boundary (or away from it), the chiral ABP will be able to detach from the confining wall more difficultly (or more easily) than a standard ABP with similar self-propulsion speed *v*.

The Chiral ABP also improves the representation of the survival strategies. First of all, the regions with high survival-path probability density are broader than in the simple ABP model and display bent ellipsoid shapes (particularly evident, e.g. in Fig. 2i). In addition, also the location of the high-density region in panel (b) is correctly reproduced in panel (h). However, at least within the range of parameters we explored in this work, the chiral ABP does not fully capture the wide delocalization of the survival-path probability and the presence of concentric high-density rings. Both these features reflect the occurrence of very long circular trajectories the type shown in Fig. 3c.

In spite of this limitation, this analysis illustrates well how the study of the survival transition-path density can offer insight to improve on a statistical physics model for the dynamics of a specific active agent.

At the same time, this comparison illustrates that the information that can be assessed by considering the survival-path density alone is partial, since it does not enable us to predict the probability of surviving starting from a given initial condition, nor does it yield the order of events that characterize a successful survival strategy.

#### Committor function

**Figure 4:**
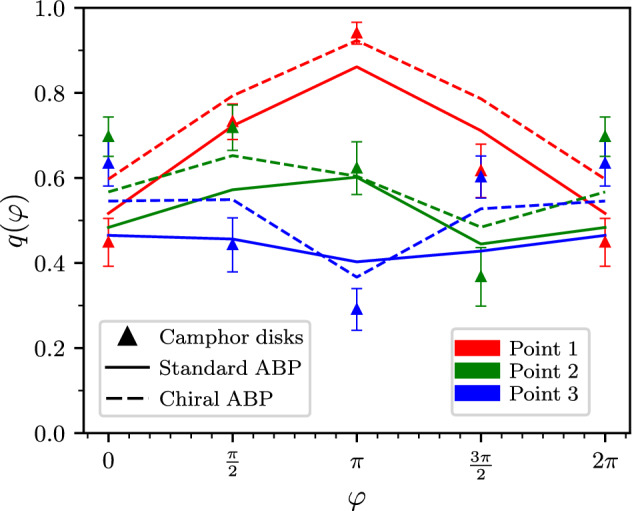
Committor *q* as a function of the exitangle $\varphi$ for the three different initial conditions. Mean and standard errors of camphor-disk experiments are given by the symbols and the associated vertical bars respectively. Solid lines represent the results of the plain ABP (with a persistence length $\ell = 0.2$, see “Materials and methods” section) and dashed line are the results of the chiral ABP.

To complement the information provided by the survival probability density and obtain a more complete characterization of the survival strategies, we used the same simulation and experimental set-ups to compute the committor function for the same set of initial conditions. This time, however, we do not marginalize over the initial angle $\vartheta$, thus also investigating how the starting direction of the self-propulsion affects the chance of survival (Fig. 4). Since camphor disks do not have an inner identifiable angle variable $\vartheta$, in the following discussion we will consider as initial angle for the process the exit angle $\varphi$, as obtained by comparing the position of the particles in the trajectory frames registered just before and just after the particle leaves one of the three circular regions 1–3. For consistency, by considering different integration time steps as the equivalent of experimental frames, the same procedure is used to measure the exit angle $\varphi$ of the ABPs.

We stress the fact that, in the limit in which the circular regions 1, 2, and 3 associated with the initial conditions have negligible size, the committor of a passive Brownian particle does not depend on $\varphi$. Hence, in this limit, any modulation in the committor *q* with the exit angle $\varphi$ represents a clear signature of the particle’s activity. In practice, to accumulate significant statistics the three circular regions where the initial position is located must have finite size (see Fig. 1). However, we checked that this finite-size effect introduces only a very weak modulation with $\varphi$, much smaller than the contribution coming from the particle’s activity (see Fig. S4 in SI).

In Materials and Methods, we discuss the partial differential equation obeyed by the committor for an ABP and in the SI we provide a numerical scheme to solve this equation for low-dimensional systems.

In Fig. 4 we compare the experimental results for $q({\varvec{r}}_a,{\varphi })$ with the predictions obtained in the standard and in the chiral ABP models. In both models and in experiment, the committor function shows a strong modulation with $\varphi$, with a similar qualitative behavior. This suggests that the general properties of the committor function do not depend on the details of the self-propulsion mechanism. Some of these features are in fact easy to interpret: for instance, the value of the committor always marks a drop when the exitangle heads directly toward the death region $\text {R}$. Some other features, on the other hand, reflect a more complex underlying mechanism: For example, in the cases of particles exiting from point 3, the committor reach their maximum when the exit angle points in the direction opposite to the target. The reason for this counter-intuitive fact is that particles can efficiently reach the target following the “shuttle” strategy, i.e. by first traveling to the boundary of the disk and then slide along it, till they reach the target zone. In the standard ABP, this effect increases with the persistence length of the particle (see Materials and Methods), leading to a general increase of the committor function for all three points considered (see Fig. S5, in SI).

Another interesting feature of our results emerges from observing that the experimentally measured committor associated with point 2 is largest when the camphor disk leaves region 2 heading “North-East” ($\varphi \in [0,\pi /2]$). However, the survival-path density is maximum in the region located at “North-West”, relatively to the initial condition (see Fig. 2b). This feature clearly illustrates how the information encoded in the committor *q* and the survival-path density $m_s$ is not trivially correlated. Indeed, the camphor disks that survive by crossing the “North-West” region tend to explore this part of the disk for a relatively long time, before they can finally reach the safe zone, hence the relatively large surviving probability density of this area. On the other hand, the disks that leave point 2 heading “North-East” have the largest probabilty to reach the safe zone, because they tend to hit the border of the disk and then rapidly slide toward the safe zone via the “shuttle” mechanism.

We have found that the standard ABP model is able to pinpoint some non-trivial qualitative feature of the committor, arising from the interplay between the activity of the particles and its environment. The agreement with the experimental results is, however, significantly improved using the chiral ABP. In particular, the addition of the angular drift provides a modulation of the committor curve of point 2 that follows closely the one observed in the experiments, with a maximum survival-path probability for an exit angle of $\pi /2$ rather than $\pi$.

Finally, a remark about Fig. 4 is in order: The error bars of experimental data represent the statistical error as obtained from the various experimental trajectories starting from one of the given initial points 1–3. However, these trajectories were recorded during only 5 different experimental runs (see SI) and in each run, the defects in the shape of the disk might induce the particle to have a clockwise or counterclockwise rotation lasting throughout the entire run. Therefore the number of clockwise trajectory slices and counterclockwise slices are not evenly distributed in the dataset, which could generate some additional asymmetry in the data. For example, the blue experimental curve, which is expected to be symmetric, is instead displaying some asymmetry even beyond the error bars.

## Conclusions

Considering non-equilibrium dynamics as the most general and distinctive feature of life, here we have shown for the first time that the framework of Transition Path Theory^20–22^ can be successfully applied to investigate the survival behavior of active agents. In particular, we showed how powerful concepts such as the reactive probability density and the committor function provide important and complementary insight on survival and target-search strategies of camphor disks and ABPs in a Petri dish. While the survival-probability density is large in the regions that are most visited by a particle escaping a death zone and ultimately reaching a safe zone, the committor function expresses by definition the survival probability of a particle as a function of its micro-state

By inspecting this information and comparing the experimental trajectories of camphor disks with the stochastic simulations of ABPs, similar and general features of survival strategies for the considered system emerge. These include a qualitatively similar dependence of the surviving odds on the initial direction of motion and a prominent “shuttle” effect consisting in navigating along the boundaries of the confining environment to reach the target more efficiently. This surviving mechanism is quite expected for ABP particles, which tend to slide along steep walls because of the large persistence of their motion.

Furthermore, an analysis of the transition-path density and of committor function may directly suggest possible modeling improvements. In our case, it showed how the similarity between experiments and simulations is increased once a chiral ABP model is adopted instead of the standard ABP.

We emphasize that, in the ABP models we adopted in this work, the time scale for fuel consumption and waste production are assumed to be infinitely longer than those associated with the intrinsic stochastic dynamics of the Brownian particle. In this asymptotic regime, the committor and transition path density do not explicitly depend on time, reflecting the fact that the survival strategy of the active particle is not adapting to the changes in the environment it contributed to generate (e.g. by depositing waste or consuming fuel). A possible direction of improvement of the present theoretical description would be to introduce history-dependent biasing forces to mimic the effect of waste production and fuel consumption. If the characteristic time scales associated with the dynamics of these biases is chosen to be comparable with that of the diffusive dynamics of the active Brownian particle, then the committor and transition path density will develop an explicit time dependence. In this case, the time evolution of these quantities would reflect the adaptation of the survival strategy, in response to the change of the environment.

Our work sets the stage for a even more qualitative and quantitative research on fundamental questions regarding survival and the definition of life in general. The statistical mechanical approach adopted in this study can be applied to several other systems. Examples include bacteria foraging nutrients or escaping from toxic substances^34–37^, nanoparticles acting as drug delivery agents^38–41^, sperm cells navigating towards the egg^42^, and autonomous robots.

This study provides a link between chemical equilibrium and emergent behavior by way of the committor. In this way we hope that such fundamental yet elusive biological concepts such as the thrive for survival can be quantitatively characterized using this or a similar framework. This will advance not only our designs of future technologies but deepen our understanding of living systems.

## Materials and methods

### Experimental setup

The experiment consists of a camphene–camphor–polymer self-propelled disk floating on a water surface and exploring a confining environment provided by a Petri dish with a radius $\sigma$ centered in the origin. As depicted in Fig. 1, the reactant region is selected as the circular region located at the center of the dish with a radius of $r_{\text {R}} = 0.1 \sigma$ and the T region as the circular segment characterized by $x_{\text {T}}<-\, 0.8\sigma$. We select three non-overlapping circular regions with radius $r_{c} = 0.1\sigma$, numbered from 1 to 3, as the points where the committor function is evaluated, see Fig. 1. We then compute the committor in the three points as a function of the exitangle $\varphi$ defined as the angle with which the searcher leaves the circular region under consideration and computed using the particle position in the first and the last trajectory frames in which the particle is observed to be out and in the circle, respectively. The exitangle $\varphi$ is then discretized in four angular slices: a slice with $-\pi /4 \le \vartheta < \pi /4$ labeled equivalently as $\varphi =0$ or $\varphi =2\pi$, a slice with $\pi /4 \le \vartheta < 3\pi /4$ labeled as $\varphi =\pi /2$, a slice with $3\pi /4 \le \vartheta < 5\pi /4$ labeled as $\varphi =\pi$, and a slice with $5\pi /4 \le \vartheta < 7\pi /4$ labeled as $\varphi =3\pi /2$.

### Active particle experiments

#### Materials and instrumentation

Digital camera, LED flat panel, Petri dishe (Ø= 9 cm), 4 mm pill press, nitrile gloves, hot plate with magnetic stirrer, 100 ml beaker, glass cover, magnet, 50 ml flat beaker, nitrile rubber sheet *Chemicals:* (1R)-(+)-camphor (98% purity, CAS: 464-49-3, Sigma-Aldrich), camphene (95% purity, CAS: 79-92-5, Sigma Aldrich), Polyisobutylene (PIB, CAS:9003-07-0, Sigma Aldrich), MilliQ purified water.

#### Active particle preparation and observation

Camphene, camphor and PIB were weighed off at the desired weight ratio (45% camphene, 45% camphor and 10% PIB) and added to a beaker containing a stirrer magnet. The beaker was then covered with a glass cover and placed on a hot plate set to 180 $^\circ$C. Once some of the camphene had melted, the magnetic stirrer was activated and left stirring until all of the camphor and PIB had been dissolved in the liquid camphene. The liquid mixture was then transferred into a flat beaker lined with a sheet of nitrile rubber (harvested from a glove) to prevent the material from sticking to the glass. Once solidified, the material can be stored in an airtight container or in the freezer to prevent the volatile components (camphene and camphor) from evaporating.

For active particle preparation, the material was pressed into a disk-shaped pill. The disk was then placed onto a ca. 0.4 mm thick water layer inside a Petri dish with a diameter of either 12 or 19.5 cm. The ratio between pill and dish diameter was kept at 1:30. The Petri dish was illuminated from below with an LED panel to improve contrast. Footage was obtained using a top mounted camera. All experiments were performed at room temperature ($22 \pm 2\,^\circ$C).

Two types of cameras were used to obtain video footage of the active particles. We used either a NEX VG20EH from SONY (25 fps) or a Logitech C920 Webcam (30 fps). Footage was either saved on the camera on SD-card or directly recorded to a laptop.

To process the data, raw footage was concatenated, cropped and compressed to .mkv format using the FFMPEG distribution. FFMPEG was also used to edit the footage in any additional way as well as to extract individual frames from the footage. Additional image processing to maximize contrast for each frame and extracting positions of the object was done using the ImageJ software^43^. The resulting dataset contains active particle positions ($x_n$ and $y_n$) for all *n* frames.

### Stochastic models of active motion

#### Standard ABP model

The conventional ABP model describes the stochastic dynamics of an active particle characterized by a self-propulsion speed along a direction $\vartheta$ that evolves according to a rotational diffusion process: In two-dimensions, the equations of motion in the Itô discretization form read:

2a $$\begin{aligned} \varvec{r}_{i\!+\!1}= & {} \varvec{r}_{i} + v \varvec{u}_{i} \, \Delta t - \mu \varvec{\nabla } U(\varvec{r}_{i}) \Delta t + \sqrt{2D\Delta t} \, \varvec{\xi }_i, \end{aligned}$$

2b $$\begin{aligned} \vartheta _{i\!+\!1}= & {} \vartheta _{i} + \sqrt{2D_{\vartheta }\Delta t} \, \eta _i. \end{aligned}$$

Here, $\Delta t$ is the integration time step, $\varvec{r}_{i} = (x_{i},y_{i})$ denotes the position at time $t_i= i\Delta t$, and $\varvec{u}_{i} = (\cos {\vartheta _{i}},\sin {\vartheta _{i}})$ is the instantaneous orientation of the self-propulsion speed *v*. The external potential $U(\varvec{r})$ has been introduced to model possible obstacles in the environment that can hinder navigation. Finally, $\mu$ is the effective mobility of the agent, *D* and $D_{\vartheta }$ represent the translational and rotational diffusion coefficients respectively, and the components of $\varvec{\xi }_i = (\xi _{x,i},\xi _{y,i})$ and $\eta _{i}$ are independent centered Gaussian random variables with unit variance. We note that, in the limit of null drift velocity, the equations of motion (2a) and (2b) decouple and the stochastic dynamics reduces to the standard (i.e. passive) Brownian motion.

#### Chiral ABP model

As a generalization of the ABP, we also introduce a drift term in the evolution of the directional angle. Namely, we consider the chiral ABP^44^ of which the ABP is a special limiting case obtained by setting the angular drift term to zero. In this case, the discretized equations of motion are read:

3a $$\begin{aligned} \varvec{r}_{i\!+\!1}= & {} \varvec{r}_{i} + v\, \varvec{u}_{i} \, \Delta t - \mu \varvec{\nabla } U(\varvec{r}_{i}) \Delta t + \sqrt{2D\Delta t} \, \varvec{\xi }_i, \end{aligned}$$

3b $$\begin{aligned} \vartheta _{i\!+\!1}= & {} \vartheta _{i} + \omega \Delta t + \sqrt{2D_{\vartheta }\Delta t} \, \eta _i. \end{aligned}$$

#### Simulation setup

Since the motion of the camphor disks used in experiments is not significantly affected by translational diffusion, we set $D = 0$ also in our ABP and chiral ABP simulations. Then, the characteristic time scale is determined by $\tau =1/D_{\vartheta }$, while the natural length scale is given by the radius of the Petri dish $\sigma$. For reference simulation of passive Brownian particles we again integrated Eq. (2a) by setting $v=0$ and $D=\sigma ^2/\tau$.

We modeled the confining boundary of the Petri dish using a circularly symmetric quartic potential $U(x,y) = (x^{2}+y^{2})^{2}$ which acts on the agent only if it is closer than $0.01\sigma$ to the boundary. The mobility $\mu$ couples the external potential to the motion of the particle, thus $\sigma ^2/\tau \mu$ can be seen as an energy scale. We emphasize that, while for the passive Brownian particle Einstein’s relation ($D / \mu = k_B T$) holds, for an ABP, any connection to the thermal energy $k_BT$ is lost since active particles do not obey fluctuation-dissipation theorem.

Consequently, only two single dimensionless parameters are left for the problem: the persistence $\ell = v\tau /\sigma$, which corresponds to the persistence of motion when the angular drift is set to zero, and the quality factor^45^ $M=\omega \tau /2\pi$, which measures the importance of the angular drift term. Conventionally, the activity of an active particle is measured by the Péclet number $\text {Pe}$, a parameter measuring the importance of the motion due to the self propulsion with respect to the motion of a purely passive reference particle. In our case, $\text {Pe}=v \tau / \sigma$ coincides with the persistence.

In this paper, we consider the cases of a standard ABP ($\omega =0$) with $\text {Pe} = 0.2$ and $\text {Pe} = 0.4$. For the simulation of chiral ABPs, at the beginning of each new trajectory, $\text {Pe}$ is extracted from a uniform distribution in between 2.4 and 4.4 and the quality factor *M* is extracted from a Gaussian distribution with zero mean and variance equal to 1.6. Such distributions have been chosen to qualitatively maximize the similarity between the experimental results and the simulations.

For each circular point, we simulate $10^{4}$ trajectories starting from the point with a random orientation angle $\vartheta$ and ending when they either reach the target or the reactant. This is enough to ensure that the error of the mean of the simulated committor is below $10^{-2}$ in all cases.

#### Committor function of an ABP solves modified Backward Kolmogorov equation

In the following, we focus on the standard ABP description of active motion and derive a partial differential equation which enables to numerically compute it using standard finite difference methods.

We consider the case in which the ABP is in some initial condition $\Gamma (0) = (\varvec{r}_0, \vartheta _0)$ with the position ${\varvec{r}}$ placed outside $\text {R}$ and $\text {T}$. In this case, the probability of long-time survival corresponds to the odds that the agent reaches $\text {T}$ before entering $\text {R}$. The survival probability coincides with the definition of the committor function when $\text {R}$ is identified with a reactant state and $\text {T}$ with a target (product).

In the limit of vanishing self-propulsion speed, the ABP equations of motion reduce to a conventional overdamped Langevin equation. In this case, the committor function is mathematically equivalent to the solution of the problem:

4 $$\begin{aligned} \hat{H}^\dagger ~ q({\varvec{r}})= & {} 0,\quad \text {subject to } \quad {\left\{ \begin{array}{ll}\left. q(\varvec{r})\right| _{\varvec{r} \in R}=0\\ \left. q(\varvec{r})\right| _{\varvec{r} \in T}=1 \end{array}\right. }, \end{aligned}$$

where the operator $\hat{H}^\dagger := {-}D \left( \varvec{\nabla }^2 - \frac{1}{k_BT} \varvec{\nabla }U({\varvec{r}}) {\cdot \varvec{\nabla }} \right)$ is the adjoint of the Fokker-Planck operator $\hat{H}$^46^ (also referred to as the Backward Kolmogorov operator).

Let us now derive the equivalent of Eq. (4) for an ABP. To this end, we consider the propagator, i.e. the conditional probability $\mathbb {P}(\Gamma ,t|\Gamma _0)$, for the particle to be in $\Gamma =(\varvec{r},\vartheta )$ at lag time *t* given it was at $\Gamma _0 = (\varvec{r}_0,\vartheta _0)$ at time $t=0$ (see Supplemental Material of Ref.^15^ for an explicit expression of this propagator). In the region outside $\text {R}$ and $\text {T}$ (where the fuel concentration is uniform) and for $t>0$, $\mathbb {P}(\Gamma ,t|\Gamma _0)$ obeys the Fokker-Planck equation associated with the stochastic equations of motion (2a) and (2b):

5 $$\begin{aligned} -\partial _t \mathbb {P}(\Gamma , t| \Gamma _0) {=} \sum _{i=x,y}\partial _i \big (v \varvec{u}_i - D \partial _i - \mu \partial _i U(\varvec{r})\big ) \mathbb {P}(\Gamma , t| \Gamma _0) -D_{\vartheta } \partial _\vartheta \partial _{\vartheta } \mathbb {P}(\Gamma , t| \Gamma _0), \end{aligned}$$

in short, $-\,\partial _t \mathbb {P} = \hat{\mathcal {H}} \mathbb {P}$. It is convenient to recast this expression in the form of a continuity equation:

6 $$\begin{aligned} -\partial _t \mathbb {P}(\Gamma , t| \Gamma _0)= & {} \sum _{\mu =x,y,\vartheta } \partial _\mu J_\mu (\Gamma , t| \Gamma _0). \end{aligned}$$

Here, $J_{i}$ and $J_\vartheta$ are the spatial and angular components of the Fokker–Planck current, respectively:

7a $$\begin{aligned} J_{i}(\Gamma , t| \Gamma _0)= & {} \big [v \varvec{u}_i - D \partial _i - \mu \partial _i U(\varvec{r})\big ] \mathbb {P}(\Gamma , t| \Gamma _0) \;, \end{aligned}$$

7b $$\begin{aligned} J_{\vartheta }(\Gamma , t| \Gamma _0)= & {} - D_{\vartheta } \partial _{\vartheta } \mathbb {P}(\Gamma , t| \Gamma _0). \end{aligned}$$

To obtain an explicit expression for the committor function $q(\Gamma )$ for an ABP, we introduce a modified propagator $\mathbb {P}^{\partial \text {W}}(\Gamma ,t|\Gamma _0)$, which expresses the probability of performing a transition from $\Gamma _0$ to $\Gamma$ in time *t* under the constraint of not having entered neither the region $\text {R}$ nor $\text {T}$ (in the following region $\text {W}:= \text {R} \cup \text {T}$) before time *t*. In the SI we show that, in the region outside $\text {W}$, $\mathbb {P}_{\partial \text {W}}$ and $\mathbb {P}$ obey the same Fokker-Planck equation Eq. (5). In turns, $\mathbb {P}^{\partial \text {W}}$ defines a modified probability current $J^{\partial \text {W}}_{\nu }$ through Eqs. (7a) and  (7b).

We consider an explicit mathematical expression for the definition of the committor function, obtained by integrating over the time interval *t* the flux of the modified probability current $J^{\partial \text {W}}_{\nu }$ through the boundary of region $\text {T}$:

8 $$\begin{aligned} q(\varvec{r}_0,\vartheta _0) = - \sum _{i=x,y}\int _{0}^{\infty } {\textrm{d}}t \int {\textrm{d}}\vartheta^{\prime} \int _{\partial {\text{T}}} {\textrm{d}}\sigma ({\varvec{r}^{\prime}})\, \hat{n}_i({\varvec{r}^{\prime}})\, J_{\partial {\text {W}}}^{i} (\varvec{r}^{\prime},\vartheta ^{\prime},t|\varvec{r}_0,\vartheta _0), \end{aligned}$$

where $d\sigma ({\varvec{r}'})$ is an infinitesimal hyper-surface at ${\varvec{r}'} \in \partial \text {T}$ and $\hat{n}_i({\varvec{r}'})$ is the *i*-th component of an oriented three-dimensional versor, orthogonal to the surface $\partial \text {T}$, at the point ${\varvec{r}'}$.

In the SI, we show that starting from this equation one obtains the generalization of Eq. (4) to the case of active Brownian dynamics. The associated partial differential equation obeyed by the committor $q(\Gamma )$ corresponds to the static Backward Kolmogorov equation associated with the Fokker–Planck operator $\hat{\mathcal {H}}$, i.e.

9 $$\begin{aligned} \hat{\mathcal {H}}^\dagger q(\Gamma ) = 0. \end{aligned}$$

In two dimensions, this equation can be very efficiently solved with a finite-difference algorithm (see also SI). Equivalently, the committor function can also be estimated using stochastic methods, i.e. by integrating the equations of motion (2a) and (2b) and then counting how many times the particle reaches $\text {T}$ before visiting $\text {R}$.

The possibility of computing the committor function does not only enable us to predict the survival probability as a function of the initial condition $\Gamma _0$, but also provides crucial information to decode the survival strategy. Indeed, in the statistical mechanical description of rare events, iso-committor hyper-surfaces are commonly employed to measure the advancement of the transition along an optimal reaction coordinate^29,30^. The concept of reaction coordinate is strictly applicable only to microscopically reversible systems undergoing spontaneous transitions. Notwithstanding this, the iso-committor surfaces associated with our ABP problem still encode the information about the optimal survival strategy. To see this, let us focus on survival trajectories of agents placed at the border of the death region $\text {R}$. Surviving agents are those that manage to move away from $\text {R}$ and reach the safe zone $\text {T}$. In this case, propagating toward the target by orthogonally piercing iso-committor surfaces represents the most convenient option, since at each step the chance of survival is maximally increased.

## Supplementary Information

Supplementary Information.

## Data Availability

The datasets used and/or analysed during the current study available from the corresponding author on reasonable request.
