# Supplementary material for: Survival strategies of artificial active agents
Source: Sci Rep. 2023 Apr 6;13:5616. doi: 10.1038/s41598-023-32267-3 (PMC10079664; doi:10.1038/s41598-023-32267-3)
Supplement: Supplementary file 1 — Supplementary Information. [file 41598_2023_32267_MOESM1_ESM.pdf]

Supporting Information

Luigi Zanovello[1,2], Richard J. G. Löffler[3], Michele Caraglio[2], Thomas Franosch[2], Martin M. Hanczyc[3,4,*], and Pietro Faccioli[1,5,]**

[1]Physics Department, University of Trento, Via Sommarive 14, Povo, 38123, Trento, Italy
[2]Institut für Theoretische Physik, Universität Innsbruck, Technikerstraße 21A, A-6020, Innsbruck, Austria
[3]Department of Cellular, Computational and Integrative Biology (CIBIO), University of Trento, Via Sommarive 9 Povo, 38123, Trento, Italy
[4]Department of Chemical and Biological Engineering, University of New Mexico, Albuquerque (NM) 87106, USA
[5]Trento Institute for Fundamental Physics and Applications (INFN-TIFPA), Via Sommarive 14 Povo, 38123, Trento, Italy
[**]pietro.faccioli@unitn.it
[*]martin.hanczyc@unitn.it

ABSTRACT

In this Supporting Information, we provide mathematical details about the theory of the committor function in the conventional and chiral Active Brownian Particle (ABP) model and we discuss numerical methods to compute it. We also provide information about our analysis of experimental and simulation data.

1 Theory

1.1 The ABP committor satisfies the backward Kolmogorov equation

The goal of this section is to show that, in analogy with passive systems, the committor for an ABP obeys a Backward Kolmogorov equation,

$$\hat{\mathscr{H}}^{\dagger} q(\Gamma) = 0\,, \tag{1}$$

where $\hat{\mathscr{H}}^{\dagger}$ is the active generalization of the Backward Kolmogorov operator:

$$\hat{\mathscr{H}}^{\dagger} = \sum_{i=x,y} \big(\mu \partial_i U(\boldsymbol{r}) - D\partial_i - v\boldsymbol{u}_i\big)\partial_i - D_{\vartheta}\partial_{\vartheta}^2\,. \tag{2}$$

To prove this result, we begin by introducing a modified propagator $\mathbb{P}^{\partial \mathrm{W}}(\Gamma, t|\Gamma_0)$ which represents the probability for an ABP to go from Γ_0 to Γ in time t under the constraint of never entering the region W. To provide an explicit path integral representation of this propagator, we introduce a characteristic function, $\Omega_{\mathrm{W}}(\boldsymbol{r}', \vartheta')$ that is 0 if the particle is outside region W (where $\mathrm{W} = \mathrm{R} \cup \mathrm{T}$) and is very large otherwise. According to this definition, the contribution to the path integral coming from trajectories that touch W is strongly suppressed. The path integral representation of the modified propagator then reads (see Supplemental Material of Ref. 1 for the standard propagator) :

$$\mathbb{P}^{\partial \mathrm{W}}(\Gamma, t|\Gamma_0) = \mathscr{Z}^{-1} \int \mathscr{D}\boldsymbol{r}' \int \mathscr{D}\vartheta'\, e^{-\frac{1}{4D_{\vartheta}} S_{\mathrm{rot}}[\vartheta'] - \frac{1}{4D} S_{\mathrm{trans}}[\boldsymbol{r}', \vartheta'] - \int_0^t d\tau \Omega_{\mathrm{W}}[\boldsymbol{r}'(\tau), \vartheta'(\tau)]}, \tag{3}$$

where $\mathscr{Z}$ is a normalization constant and:

$$S_{\mathrm{trans}}[\boldsymbol{r}', \vartheta'] = \int_0^t \mathrm{d}\tau \Big[\dot{\boldsymbol{r}}'(\tau) - v\boldsymbol{u}'(\tau) + \mu \nabla U\big(\boldsymbol{r}'(\tau)\big)\Big]^2\,, \tag{4}$$

$$S'_{\mathrm{rot}}[\vartheta'] = \int_0^t \mathrm{d}\tau \big[\dot{\vartheta}'(\tau)\big]^2\,. \tag{5}$$

Since $\mathbb{P}(\Gamma, t|\Gamma_0)$ and $\mathbb{P}^{\partial \mathrm{W}}(\Gamma, t|\Gamma_0)$ differ only from the presence of the time integral of the characteristic function $\Omega_{\mathrm{W}}(\boldsymbol{r}', \vartheta')$ along the path, by imposing the condition $\Omega_{\mathrm{W}}(\boldsymbol{r}, \vartheta)\mathbb{P}^{\partial \mathrm{W}}(\Gamma, t|\Gamma_0) = 0$, one has that $\mathbb{P}(\Gamma, t|\Gamma_0)$ and $\mathbb{P}^{\partial \mathrm{W}}(\Gamma, t|\Gamma_0)$ are solution to the same FP equation in the region outside W.

The modified propagator can be used to provide an explicit definition of the committor function of an ABP:

$$q(\boldsymbol{r},\vartheta) = \sum_{i=x,y} \int_0^\infty \mathrm{d}t \int \mathrm{d}\vartheta' \int_{\partial \mathrm{T}} \mathrm{d}\sigma(\boldsymbol{r}')\, \hat{n}_i(\boldsymbol{r}')\, J^i_{\partial \mathrm{W}}(\boldsymbol{r}',\vartheta',t|\boldsymbol{r},\vartheta). \tag{6}$$

Let us now apply the Backward Kolmogorov operator to this definition of active committor:

$$\begin{aligned}
\hat{\mathscr{H}}^\dagger q(\boldsymbol{r},\vartheta) &= -\hat{\mathscr{H}}^\dagger \sum_{i=x,y} \int_0^\infty \mathrm{d}t \int \mathrm{d}\vartheta' \int_{\partial \mathrm{T}} \mathrm{d}\sigma(\boldsymbol{r}')\, \hat{n}_i(\boldsymbol{r}')\, J^i_{\partial \mathrm{W}}(\boldsymbol{r}',\vartheta',t|\boldsymbol{r},\vartheta) \; = \\
&= \hat{\mathscr{H}}^\dagger \sum_{i=x,y} \int_0^\infty \mathrm{d}t \int \mathrm{d}\vartheta' \int_{\partial \mathrm{T}} \mathrm{d}\sigma(\boldsymbol{r}') \left[\hat{n}_i(\boldsymbol{r}') \left[D\partial_i' - v\boldsymbol{u}_i' + \mu \partial_i' U(\boldsymbol{r}')\right] + \hat{n}_\vartheta(\boldsymbol{r}') D_\vartheta \partial_\vartheta'\right] \mathbb{P}^{\partial \mathrm{W}}(\Gamma',t|\Gamma) = \\
&= \sum_{i=x,y} \int_0^\infty \mathrm{d}t \int \mathrm{d}\vartheta' \int_{\partial \mathrm{T}} \mathrm{d}\sigma(\boldsymbol{r}') \left[\hat{n}_i(\boldsymbol{r}') \left[D\partial_i' - v\boldsymbol{u}_i' + \mu \partial_i' U(\boldsymbol{r}')\right] + \hat{n}_\vartheta(\boldsymbol{r}') D_\vartheta \partial_\vartheta'\right] \hat{\mathscr{H}}^\dagger \mathbb{P}^{\partial \mathrm{W}}(\Gamma',t|\Gamma),
\end{aligned} \tag{7}$$

where ∂_i' (∂_ϑ') denotes the partial derivatives with respect to the components of the final position vector $\boldsymbol{r}'$ (final drift angle ϑ'), and $\boldsymbol{u}'$ is the drift direction defined in terms of ϑ'. On the other hand, the Backward Kolhmogorov operator acts on the initial state variables, $\Gamma = (\boldsymbol{r},\vartheta)$.

We now recall that the ABP stochastic differential equations define a Markovian dynamics in the extended space spanned by the state variable $\Gamma = (\boldsymbol{r},\vartheta)$. Then, in analogy with the passive case, for a time homogeneous system we have

$$\partial_t \mathbb{P}^{\partial \mathrm{W}}(\Gamma',t|\Gamma) = -\hat{\mathscr{H}}^\dagger \mathbb{P}^{\partial \mathrm{W}}(\Gamma',t|\Gamma). \tag{8}$$

Applying this result to Eq. 7 we obtain:

$$\begin{aligned}
\hat{\mathscr{H}}^\dagger q(\boldsymbol{r},\vartheta) &= -\sum_{i=x,y} \int_0^\infty \mathrm{d}t \frac{\partial}{\partial t} \int \mathrm{d}\vartheta' \int_{\partial \mathrm{T}} \mathrm{d}\sigma(\boldsymbol{r}') \left[\hat{n}_i(\boldsymbol{r}') \left[D\partial_i' - v\boldsymbol{u}_i' + \mu \partial_i' U(\boldsymbol{r}')\right] + \right. \\
&\qquad \left. + \hat{n}_\vartheta(\boldsymbol{r}') D_\vartheta \partial_\vartheta'\right] \mathbb{P}^{\partial \mathrm{W}}(\Gamma',t|\Gamma) = \\
&\qquad = \sum_{i=x,y} \int_0^\infty \mathrm{d}t \frac{\partial}{\partial t} \int \mathrm{d}\vartheta' \int_{\partial \mathrm{T}} \mathrm{d}\sigma(\boldsymbol{r}')\, \hat{n}_i(\boldsymbol{r}')\, J^i_{\partial \mathrm{W}}(\boldsymbol{r}',\vartheta',t|\boldsymbol{r},\vartheta)\,.
\end{aligned} \tag{9}$$

Let us now introduce the first passage time distribution across T, $F_{\partial \mathrm{T}}(t)$. This is defined by the flux of probability current associated with the stochastic trajectories initiated in Γ that have not entered W before time t:

$$F_{\partial \mathrm{T}}(t) = \sum_{i=x,y} \int \mathrm{d}\vartheta' \int_{\partial \mathrm{T}} \mathrm{d}\sigma(\boldsymbol{r}')\, \hat{n}_i(\boldsymbol{r}')\, J^i_{\partial \mathrm{W}}(\boldsymbol{r}',\vartheta',t|\boldsymbol{r},\vartheta)\,, \tag{10}$$

Applying this definition to Eq. (9) and performing the time integral yields

$$\hat{\mathscr{H}}^\dagger q(\boldsymbol{r},\vartheta) = F_{\partial \mathrm{T}}(t\to\infty) - F_{\partial \mathrm{T}}(t\to 0) \tag{11}$$

Finally, we assume that in a sufficiently long time, the ABP will eventually cross the boundary of W, thus $F_{\partial \mathrm{T}}(t\to\infty) = 0$. On the other hand, in the short time limit, the flux through W vanishes provided the initial state in the transition region at a finite distance from T, i.e. $F_{\partial \mathrm{T}}(t\to 0) = 0$. Then, the right-hand-side of Eq. (11) vanishes, thus implying $\hat{\mathscr{H}}^\dagger q(\boldsymbol{r},\vartheta) = 0$.

1.2 Committor for a chiral ABP and its properties

Following the same procedure outlined above, we can now derive the committor function for a chiral ABP[2]. The equations of motion for such a particle are provided by Eqs. (3a) and (3b) of the main text, therefore the Fokker-Planck equation obeyed by the chiral particle reads:

$$\begin{aligned}
-\partial_t \mathbb{P}(\Gamma,t|\Gamma_0) &= \sum_{i=x,y} \partial_i \big(v\boldsymbol{u}_i - D\partial_i - \mu \partial_i U(\boldsymbol{r})\big) \mathbb{P}(\Gamma,t|\Gamma_0) + \partial_\vartheta \big(\omega - D_\vartheta \partial_\vartheta\big) \mathbb{P}(\Gamma,t|\Gamma_0) \\
&\equiv \hat{\mathscr{H}_c}\, \mathbb{P}(\Gamma,t|\Gamma_0)\,,
\end{aligned} \tag{12}$$

where $\hat{\mathscr{H}_c}$ is the Fokker-Planck operator for a chiral ABP. This equation in continuity form reads:

$$-\partial_t \mathbb{P}(\Gamma,t|\Gamma_0) = \sum_{\mu=x,y,\vartheta} \partial_\mu J_\mu(\Gamma,t|\Gamma_0)\,, \tag{13}$$

where, once again, J_i and J_ϑ are the spatial and angular components of the Fokker-Planck current, which read:

$$J_i(\Gamma,t|\Gamma_0) = \left[v\boldsymbol{u}_i - D\partial_i - \mu\partial_i U(\boldsymbol{r})\right]\mathbb{P}(\Gamma,t|\Gamma_0)\,, \tag{14a}$$
$$J_\vartheta(\Gamma,t|\Gamma_0) = \left[\omega - D_\vartheta\partial_\vartheta\right]\mathbb{P}(\Gamma,t|\Gamma_0)\,. \tag{14b}$$

The propagator $\mathbb{P}(\Gamma,t|\Gamma_0)$ for the chiral ABP can be obtained following the procedure outlined in Ref. 3, which yields an expression analogous to the one reported in the Supplemental Material of Ref. 1:

$$\mathbb{P}(\Gamma,t|\Gamma_0) = \mathscr{Z}^{-1}\int\mathscr{D}\boldsymbol{r}'\int\mathscr{D}\vartheta' e^{-\frac{1}{4D_\vartheta}S'_{\mathrm{rot}}[\vartheta']}e^{-\frac{1}{4D}S_{\mathrm{trans}}[\boldsymbol{r}',\vartheta']}\,, \tag{15}$$

where $\mathscr{Z}$ is a normalization constant, once again:

$$S_{\mathrm{trans}}[\boldsymbol{r}',\vartheta'] = \int_0^t \mathrm{d}\tau\left[\dot{\boldsymbol{r}}'(\tau) - v\boldsymbol{u}'(\tau) + \mu\nabla U\big(\boldsymbol{r}'(\tau)\big)\right]^2\,, \tag{16}$$

and this time:

$$S'_{\mathrm{rot}}[\vartheta'] = \int_0^t \mathrm{d}\tau\left[\dot{\vartheta}'(\tau) - \omega\right]^2\,. \tag{17}$$

Consequently, similarly to the standard ABP case, the modified propagator $\mathbb{P}^{\partial\mathrm{W}}(\Gamma,t|\Gamma_0)$ can be constructed as:

$$\mathbb{P}^{\partial\mathrm{W}}(\Gamma,t|\Gamma_0) = \mathscr{Z}^{-1}\int\mathscr{D}\boldsymbol{r}'\int\mathscr{D}\vartheta'\, e^{-\frac{1}{4D_\vartheta}S'_{\mathrm{rot}}[\vartheta'] - \frac{1}{4D}S_{\mathrm{trans}}[\boldsymbol{r}',\vartheta'] - \int_0^t d\tau\Omega_{\mathrm{W}}[\boldsymbol{r}'(\tau),\vartheta'(\tau)]}\,, \tag{18}$$

which satisfies the same Fokker-Planck equation fulfilled by $\mathbb{P}(\Gamma,t|\Gamma_0)$ outside region W (Eq. 12).

This allows then for a definition of the committor analogous to the one presented in the main text for the standard ABP:

$$q(\boldsymbol{r}_0,\vartheta_0) = -\sum_{i=x,y}\int_0^\infty \mathrm{d}t\int\mathrm{d}\vartheta'\int_{\partial\mathrm{T}}\mathrm{d}\sigma(\boldsymbol{r}')\,\hat{n}_i(\boldsymbol{r}')\,J^i_{\partial\mathrm{W}}(\boldsymbol{r}',\vartheta',t|\boldsymbol{r}_0,\vartheta_0)\,. \tag{19}$$

Finally, following the same reasoning discussed in the previous section, it can be proven that also in the chiral ABP case the committor is a solution of the backward Kolmogorov equation for the system:

$$\hat{\mathscr{H}}_c^\dagger q(\Gamma) = 0. \tag{20}$$

where, this time, the backward Kolmogorov operator reads:

$$\hat{\mathscr{H}}_c^\dagger = \sum_{i=x,y}\big(\mu\partial_i U(\boldsymbol{r}) - D\partial_i - v\boldsymbol{u}\big)\partial_i - \big(D_\vartheta\partial_\vartheta + \omega\big)\partial_\vartheta\,. \tag{21}$$

2 Computing the committor from a numeric solution of the backward Kolmogorov equation

For low-dimensional systems, such as in the case of the considered models, the knowledge of the backward Kolmogorov equation can be exploited to efficiently compute the committor function using numeric methods. Since the backward Kolmogorov equation is a partial differential equation of the second order, a standard procedure that has been applied in the literature[4] relies on using a finite-difference algorithm to solve it on a grid. To use this method, one needs to specify the correct boundary conditions for the problem, which in our case can be achieved by imposing $q(\boldsymbol{r},\vartheta) = 0$ if $(\boldsymbol{r},\vartheta) \in \mathrm{R}$ and $q(\boldsymbol{r},\vartheta) = 1$ if $(\boldsymbol{r},\vartheta) \in \mathrm{T}$. Additionally, one needs to specify the boundary conditions also on the edges of the grid, which is usually achieved by considering a dimension of the grid large enough to capture the entirety of the process to then impose Neumann boundary conditions on the edges.

Here, we provide a simple validation of our theoretical derivation by comparing the value of the committor obtained by solving the backward Kolmgorov equation through a finite-difference algorithm with the one achieved through brute-force simulations, which are a less efficient way to compute it but provide a direct insight on the behavior of the system.

We consider an ABP searching for a target in an energy landscape provided by a double-well potential, similarly to the setup that we analyzed in Refs. 1 and 5. As we found in our previous studies, due to the presence of the self-propulsion the ABP presents an increased exploration capability compared to a passive particle. Thus, considering a dimension of the grid large enough along the x and y components to capture the whole process while keeping a small dimension of the grid cells

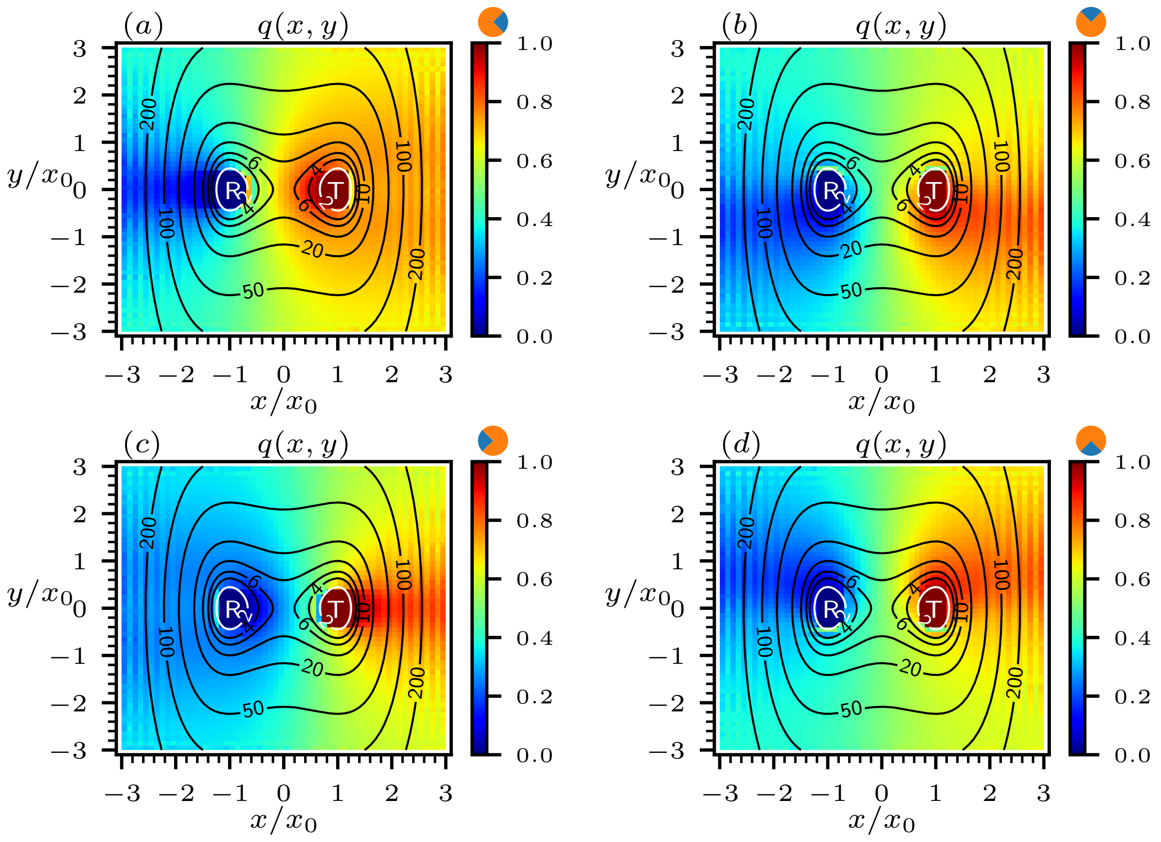

Fig. S1. Committor for an ABP in a double-well potential obtained through a finite-difference algorithm and reported as a function of the spatial coordinates x and y. The contour lines represent the underlying energy landscape with energy value reported for each contour line. The white lines with $U(x,y) = 2k_BT$ indicate the boundaries of the R and T regions. (a) Committor obtained as an average of the two angular slices located between $-\pi/4 \leq \vartheta < \pi/4$ (represented by the blue fraction of the orange pie plot on top of the bar). (b) Committor averaged between $\pi/4 \leq \vartheta < 3\pi/4$. (c) Committor averaged between $3\pi/4 \leq \vartheta < 5\pi/4$. (d) Committor averaged between $5\pi/4 \leq \vartheta < 7\pi/4$.

is computationally unfeasible, and consequently Neumann boundary conditions cannot be employed. Therefore, we resort to considering a smaller dimension of the grid and we estimate the value of the committor on its edges using brute-force simulations, to then feed this information to the finite-difference algorithm which computes the values of the committor within the grid. Notwithstanding the use of brute-force simulations on the edges, this procedure is still about one order of magnitude faster than computing the committor via brute-force simulations on the whole grid. Finally, along the ϑ component of the grid, periodic boundary conditions are used.

Following the analysis of the parameter space of this model that we provided in Ref. 5, we perform this validation employing a value of $\mu = 0.1$ for the effective mobility of the particle, we set $D = 0.1$, and we fix an energy scale for the problem as $k_BT = D/\mu$. The energy landscape is given by $U(x,y) = k_x(x^2 - x_0^2)^2 + k_y y^2/2$, where $k_x x_0^4 = 6.5k_BT$ and $k_y x_0^2 = 20k_BT$. We set the length scale for the problem as $L = k_BT/F_{\max}$, where $F_{\max}$ is the maximal value of the external force associated with the landscape along the minimum energy path linking R and T. For the considered system, we have $L \simeq x_0/10$. The time scale is instead set by $\tau = L^2/D$. The ABP has a Péclet number $\mathrm{Pe} = vL/D \simeq 9$ and a persistence $\ell^* = v/D_\vartheta L \simeq 7$. The R region for the process is determined by $U(x,y) \leq 2k_BT$ and $x < 0$, while T is identified by $U(x,y) \leq 2k_BT$ and $x > 0$. The span of the grid employed in the finite-difference algorithm is set from $-30L$ and $30L$ both along x and y, with a dimension of the cells $\delta = L$, while the grid is from 0 to 2π along the ϑ component with a dimension of the cells of $\pi/4$. Finally, the brute force simulations are performed with an integration time step of $10^{-2}\tau$ and the value of the committor for each grid point is obtained by initiating 10^3 trajectories from the center of each grid point and counting how many of them reach T first over the total.

The committor function obtained through the finite-difference algorithm (see Fig. S1) displays data in agreement with those obtained from brute-force simulations (Fig. S2), providing a validation for our theory. From these pictures, it becomes evident once more the relevance of the self-propulsion orientation on the odds of successfully finding the target. In particular, quite

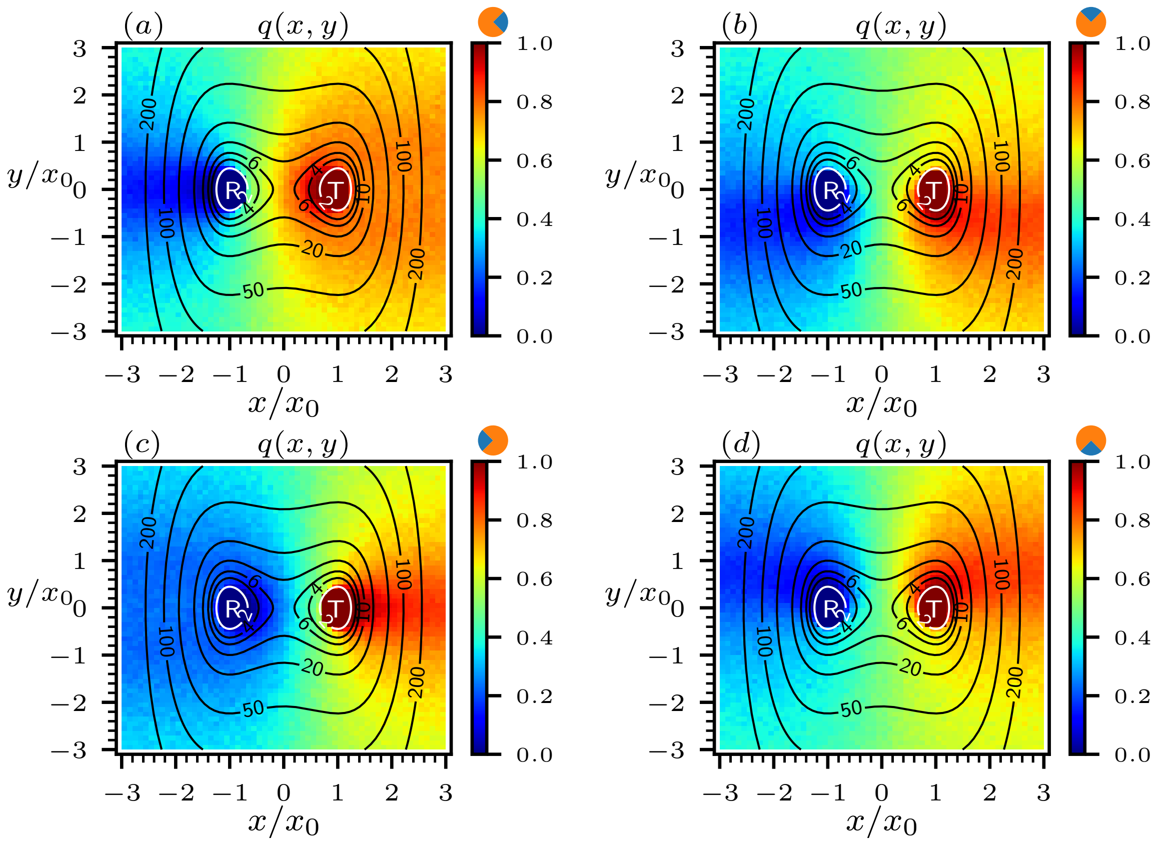

Fig. S2. Committor as in Fig. S1 but obtained from brute-force integration of the equations of motion.

intuitively, an initial self-propulsion orientation pointing towards the right leads to a much larger fraction of the configuration space with high values of the committor compared to a case with an opposite self-propulsion orientation due to the position of the target (compare Fig. S1(a) and Fig. S1(c)).

However, even though the finite-difference solution of the committor reproduces the main features of the brute-force committor, some differences are observed between the two cases. Specifically, the finite-difference solution displays a "stripe" pattern at the edges of the grid that is absent in the brute-force case. This behavior does not depend on the accuracy in the determination of the committor at the boundaries of the grid, in fact a 10 fold increase in the brute-force trajectories starting from the edges of the grid or a decrease in the simulation time step did not provide a considerable difference. Instead, the observation of this phenomenon depends on the sensitiveness of the finite-difference algorithm to the dimension of the grid cells. To properly solve the backward Kolmogorov equation on a grid, in fact, is required a smaller dimension of the grid points the more the particle moves, *i.e.* the larger the self propulsion becomes. Unfortunately, a decrease in the dimension of the grid points quickly becomes unfeasible due to the limited amount of computational resources (RAM) required to solve the equation on the grid, representing a typical "curse of dimensionality" problem. However, these small errors introduced with the finite-difference solution come with a 10 fold decrease in the computational time compared to the brute-force solution, so for some low-dimensional systems this might still be the more preferable choice.

3 Data analysis

3.1 Experimental data analysis

From the five long trajectories recorded from experiments, first the positions were obtained using ImageJ, and then the trajectory slices exiting from one of the three points and reaching R or T were extracted. For all the trajectories the positions are provided in pixels. Since the distance between the camera and the Petri dish varied across the different experiments, the dimensions and positions of the relevant regions for the study also changed during the experiments. The different data are presented in table 1, where we recall that σ is the radius of the dish, r_{R} is the radius of the R region, which has the same center of the dish, x_{T} identifies the boundary of the T region as described in the main text, and r_c is the radius of the points where the committor is calculated. Finally, we indicate with C_σ the center of the Petri dish and with C_i the center of point i. Note that the values of x_{T}

Table 1. Parameter values employed in the analysis of experimental trajectories, expressed in pixels.

Experiment	σ	C_σ	r_R	x_T	r_c	C_1	C_2	C_3
Trajectory 1	490	$(600,518)$	49	-392	49	$(-245,0)$	$(0,245)$	$(245,0)$
Trajectory 2	467	$(616,476)$	47	-374	47	$(-233,0)$	$(0,233)$	$(233,0)$
Trajectory 3	464	$(512,530)$	46	-371	46	$(-232,0)$	$(0,232)$	$(232,0)$
Trajectory 4	464	$(555,548)$	46	-371	46	$(-232,0)$	$(0,232)$	$(232,0)$
Trajectory 5	461	$(555,569)$	46	-369	46	$(-231,0)$	$(0,231)$	$(231,0)$

and C_i are provided in the reference system where the origin coincides with the center of the dish C_σ.

The experimental survival path density is computed starting from the trajectories extracted from experimental data and reaching T. The spatial positions visited during each trajectory are used to construct a frequency histogram, which is normalized by the total number of trajectory points and the area of the histogram bins. The bins were selected as squares with dimension $\delta = \sigma/20$. To construct the survival path density, only the histogram bins with every corner located inside the boundary of the Petri dish were considered.

Using the trajectory slices extracted from the experimental data, we estimate the committor in the three points $1-3$ as a function of the exit angle by counting how many trajectories $N_{\mathrm{T},i,j}$ leave point i with an angle falling in the angular slice j and then reach T, over the total number of trajectories $N_{i,j}$ leaving that point with that angular slice, therefore $q_{i,j} = N_{\mathrm{T},i,j}/N_{i,j}$. The exit angle φ is obtained from the infinitesimal displacement along the x and y coordinates between the last trajectory step within one of the points $1-3$ and the first step outside that region. We point out that some trajectory slices might be used to compute the committor in two different points, such as in the case of a trajectory leaving point 1, visiting point 2, and then reaching R or T, which in this case will be used to compute the committor both for point 1 and for point 2. Instead, trajectory slices leaving one of the three points, reentering it and then leaving it again to reach R or T will be used to compute the value of the committor in that point only once, starting from the first time they leave that region. Across the five experiments performed, a number n of about 900 trajectory slices was obtained, yielding an approximate number of 80 short trajectories for each point and angular slice.

An error for the committor is then estimated as a standard error on the mean, $\sigma_{m,i,j} = \sigma_{i,j}/\sqrt{N_{i,j}}$, where $\sigma_{i,j}$ is the standard deviation of the data:

$$\sigma_{i,j} = \sqrt{\frac{N_{\mathrm{T},i,j}(1-q_{i,j})^2 + (N_{i,j} - N_{\mathrm{T},i,j})(0-q_{i,j})^2}{N_{i,j}}}\,. \tag{22}$$

3.2 Simulation data analysis

The analysis of the simulation data follows the same pipeline outlined above, using the short trajectories slices that start from regions $1-3$ and reach R or T. The approximate number of short trajectories employed is about $2-3\cdot 10^3$ per point and angular slice, both in the case of the standard ABP and of the chiral ABP.

References

1. Zanovello, L., Caraglio, M., Franosch, T. & Faccioli, P. Target search of active agents crossing high energy barriers. *Phys. Rev. Lett.* **126**, 018001, DOI: 10.1103/PhysRevLett.126.018001 (2021).
2. van Teeffelen, S. & Löwen, H. Dynamics of a Brownian circle swimmer. *Phys. Rev. E* **78**, 020101, DOI: 10.1103/PhysRevE.78.020101 (2008).
3. Elber, R. & Shalloway, D. Temperature dependent reaction coordinates. *The J. Chem. Phys.* **112**, 5539–5545, DOI: 10.1063/1.481131 (2000).
4. Metzner, P., Schütte, C. & Vanden-Eijnden, E. Illustration of transition path theory on a collection of simple examples. *The J. Chem. Phys.* **125**, 084110, DOI: 10.1063/1.2335447 (2006).
5. Zanovello, L., Faccioli, P., Franosch, T. & Caraglio, M. Optimal navigation strategy of active Brownian particles in target-search problems. *The J. Chem. Phys.* **155**, 084901, DOI: 10.1063/5.0064007 (2021).

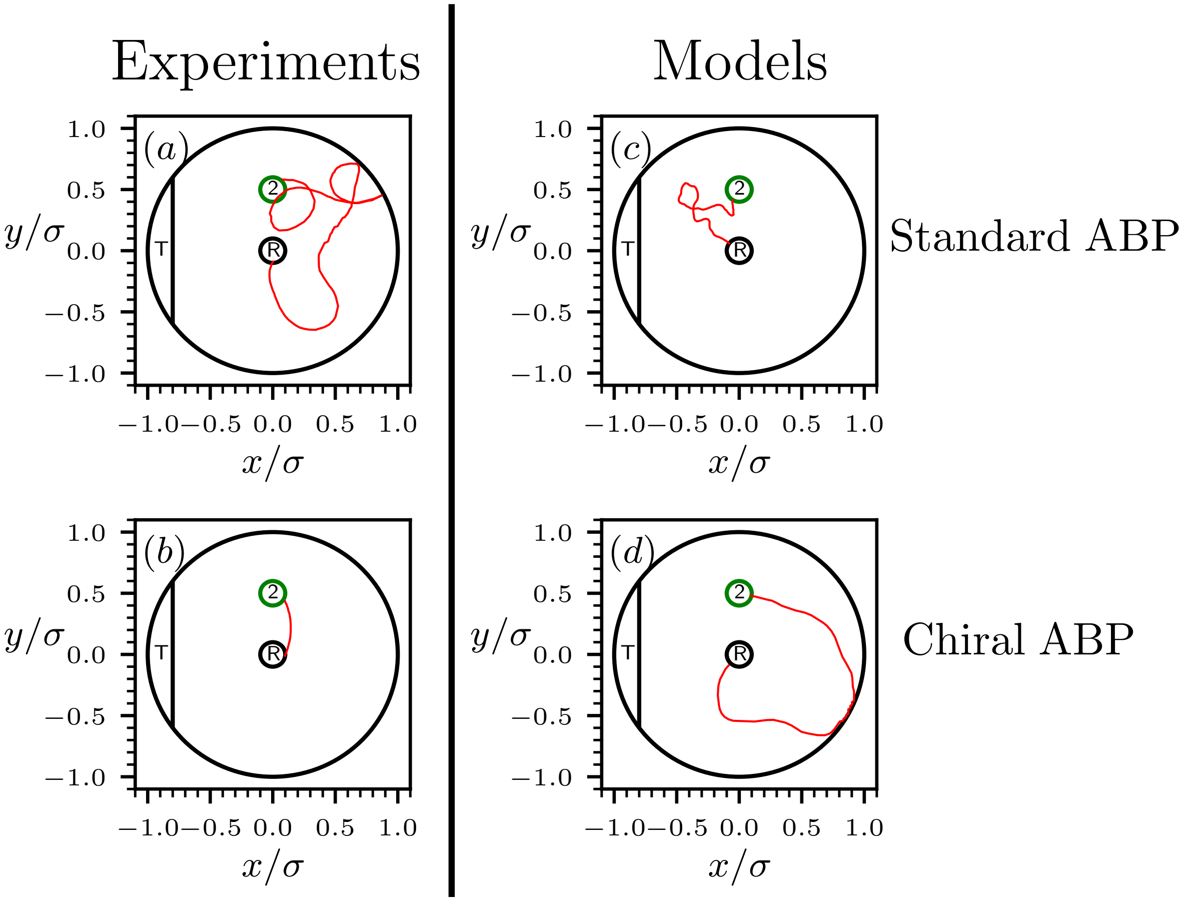

Fig. S3. (a-b) Typical experimental trajectories of a self-propelled camphor disk reaching the death region R starting from the circular region 2. (c) Typical trajectory of the standard ABP. (d) Sample trajectory of the chiral ABP.

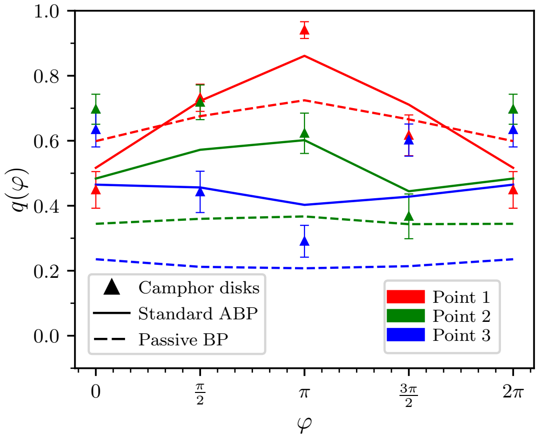

Fig. S4. Committor as a function of the exit-angle φ for a plain ABP (full lines), a passive Brownian particle (dashed lines), and a camphor disk (symbols). The observed dependency on φ in the passive case is a consequence of the finite size of the initial condition.

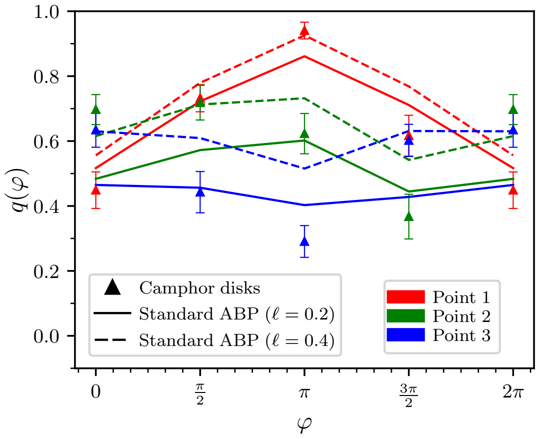

Fig. S5. Committor as a function of the exit-angle φ for an ABP with different persistence lengths (lines) and in a camphor disk experiment (symbol). The increase in the persistence length improves the agreement with the experiment.
